# Fractionated Gyroscopic Radiosurgery for Optic Nerve Glioma: A Case Report and Review of the Literature

**DOI:** 10.7759/cureus.85112

**Published:** 2025-05-30

**Authors:** Parvathi Narayanaswamy, Alisha VP, Sapna V Manocha, Ganesh Jadhav, Rohit K Chaini, Sudheer Tyagi, Saji Oommen, Sunil Chauhan, Harish Kumar, Ankit Yadav

**Affiliations:** 1 Radiation Oncology, Indraprastha Apollo Hospitals, New Delhi, New Delhi, IND; 2 Neurological Surgery, Indraprastha Apollo Hospitals, New Delhi, New Delhi, IND; 3 Medical Physics, Indraprastha Apollo Hospitals, New Delhi, New Delhi, IND

**Keywords:** fractionated stereotactic radiosurgery, ophthalmology, optic pathway glioma, paediatric oncology, zap gyroscopic radiosurgery

## Abstract

Radiotherapy offers a favorable long-term outcome for the treatment of optic pathway glioma, but at a cost of neurocognitive impairment and decreased quality of life. This has led to a chemotherapy-first approach, deferring radiotherapy till disease progression. We present a case report wherein a 20-year-old female patient with left optic nerve glioma was treated with definitive radiotherapy using a self-shielding gyroscopic radiosurgery (GRS) system. We report the first published use of GRS in the treatment of optic nerve glioma.

The patient was treated with 15 Gy at the 56% isodose line in two fractions on alternate days with 17 isocenters and 271 non-coplanar beams, amounting to a total treatment time of 63 minutes. The evaluation of the treatment plan shows considerable sparing of the normal structures, which may translate into better long-term clinical outcomes. A clinical follow-up after a month of treatment showed good clinical control without any undue treatment-related toxicity. Further follow-up is slated as per standard protocol. This case report opens up the potential for considering GRS after careful scrutiny as a first-line approach in the treatment of optic nerve glioma.

## Introduction

Optic pathway gliomas (OPGs) are low-grade gliomas that arise from the pre-cortical optic pathways. They can involve the optic nerve, optic chiasm, optic tracts, optic radiations, and the hypothalamus [1]. Gliomas affecting the optic nerve and visual pathways present in two distinct types: benign and malignant. In children, these tumors are generally benign, mostly pilocytic astrocytoma, and can be detected on orbital imaging. Optic nerve gliomas account for 2% to 5% of central nervous system tumors in the pediatric population, 1% of all intracranial neoplasms, and 7% of all gliomas [2]. These tumors can either occur sporadically or be associated with neurofibromatosis type 1 (NF1). At presentation, the majority of optic nerve gliomas are asymptomatic and are incidentally detected during screening investigations or imaging studies done for other purposes. The common presenting symptoms in benign optic nerve glioma are loss of vision, proptosis, and retro-orbital pain. Malignant optic nerve gliomas, on the other hand, clinically present with a rapid deterioration of vision, initially unilaterally and then progress to involve bilaterally. They are extremely rare and are anaplastic astrocytomas (WHO grade III) or glioblastoma multiforme (WHO grade IV) in histology. They are usually very aggressive tumors and carry a poor prognosis [3].

A suitable multidisciplinary strategy that encompasses observation, surgical intervention, chemotherapy, and radiation therapy is required for the management of optic nerve gliomas. In most patients with anterior visual pathway gliomas, clinical and radiological surveillance is favored. The role of surgery in anterior visual pathway glioma is limited and rarely done, if there is preservation of visual function and improvement in survival. For gliomas limited to the optic nerve, complete tumor resection is usually reserved for patients who have developed extreme proptosis, and is a blinding procedure. Radiation and chemotherapy are other alternatives in management when there is clinical or radiological evidence of disease progression. Radiotherapy is considered when surgical resection is not an option, and there is progressive disease following chemotherapy or a significant risk of severe visual field loss. Conventional radiation methods often target large areas of healthy brain tissue, resulting in alterations in the intellectual, emotional, and endocrine functioning. Proton beam therapy, intensity modulated photon therapy, and stereotactic radiosurgery (SRS) may reduce the dose to normal structures, thereby providing comparable tumor control outcomes with reduced late toxicities [4,5].

The prognosis for optic nerve gliomas in children is good, which is close to 100%, whereas malignant optic nerve gliomas in adults are rapidly fatal. Progressive visual decline over a period of weeks and tumor-related death typically occur within several months of onset.

## Case presentation

**Figure 1:**
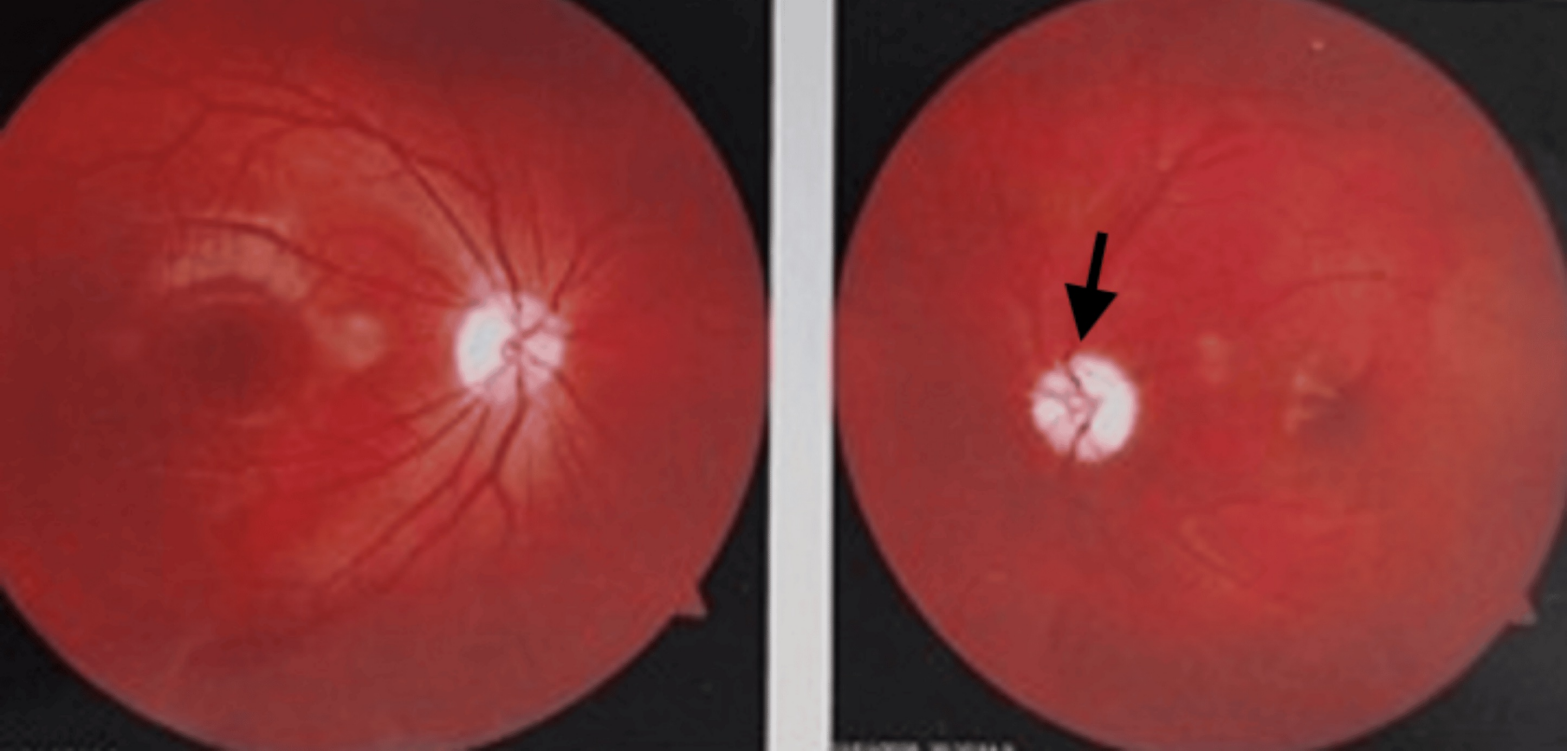
Fundus of the left eye showing pale optic disc (highlighted with arrow) and secondary optic atrophy in the left eye.

**Figure 2:**
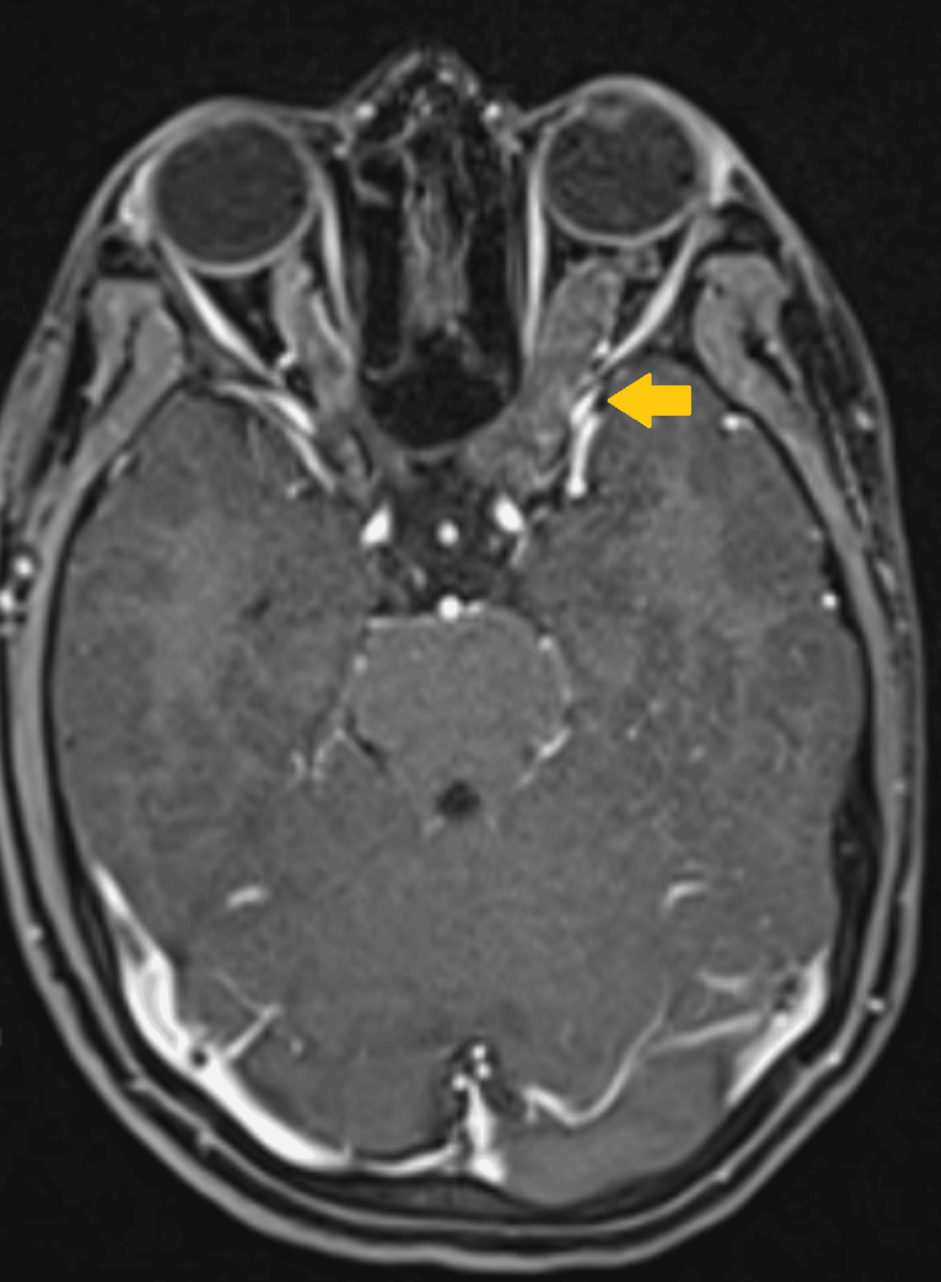
Axial section of the contrast-enhanced MRI of the brain showing fusiform enlargement of the left optic nerve (highlighted with arrow).

A 20-year-old female with no significant past medical, surgical, or family history presented to an ophthalmology outpatient department with complaints of progressively decreasing vision in her left eye of six months duration. There was no history of pain, headache, strabismus, or any other neurological symptoms or deficit. Upon clinical examination, the patient had café au lait spots all over without any other obvious stigmata associated with NF1. Ophthalmological evaluation revealed diminished vision in the left eye (hand movements +), with intact vision in the right eye (6/6 according to the Snellen chart). Posterior segment evaluation revealed a normal right eye and secondary optic atrophy in the left eye, as shown in Figure 1. Contrast-enhanced magnetic resonance imaging (CE-MRI) of the brain and orbit done on 23 March 2025 showed diffuse fusiform enlargement of the retrobulbar, intracanalicular, and cisternal parts of the left optic nerve, as illustrated in Figure 2. The lesion was T1 hypointense and T2 hyperintense. The chiasm was free. A possibility of late-onset NF1 optic nerve glioma seemed most likely.

The case was discussed in a multidisciplinary tumor board and with the patient and her family. It was decided that the patient would be treated with definitive radiotherapy. The patient and her family were counseled, and informed consent was taken. The patient was immobilized in the supine position using a headrest and thermoplastic mask. Contrast-enhanced computed tomography (CECT) of 1 mm slice thickness and CE-MRI of the brain were done for planning, followed by registration and contouring in the Brainlab iPlan Software (Brainlab AG, Munich, Germany). The gross tumor volume (GTV) was contoured, and the prescribed dose was 15 Gy to the tumor in two fractions at 7.5 Gy/Fr on alternate days, prescribed at 56% isodose. Dose constraints to all neurological structures were prescribed as per standard protocol, with special interest to chiasm, ipsilateral eye and lens, and contralateral optic nerve, and these were achieved as per international guidelines by Timmerman [6].

Fractionated gyroscopic radiosurgery (GRS) was performed on April 11th and 13th, 2025, by following standard institutional protocol for cranial robotic radiosurgery. A GRS treatment planning system (v1.8.59, ZAP Surgical Inc., San Carlos, CA) was utilized to place isocenter coordinates inside the tumor. A total of 271 non-coplanar candidate beams of diameters ranging from 4 mm to 10 mm were targeted at the isocenter coordinates. The entry and exit of beams from the contralateral eye were blocked. The weights of candidate beams were optimized with an inverse algorithm to achieve a dose distribution conformal to the tumor and a steep fall off toward the surrounding healthy tissue. Beams of low weight were discarded. A dose of 15 Gy was prescribed for the 56% isodose encompassing the tumor. Treatment was delivered on alternate days with a ZAP-X system (ZAP Surgical Inc., San Carlos, CA), which uses a compact 3 MV linear accelerator (LINAC), a collimator wheel with eight circular apertures, and kV image guidance [7]. Coupled gimbals are used to position the LINAC for non-coplanar beam delivery. Intrafraction motion of the patient was compensated by acquiring kV images every 45 seconds, deriving the offset of the patient's head by comparison with digitally reconstructed radiographs, and repositioning the patient.

A total of 17 isocenters were used to deliver 1500 cGy through 271 non-coplanar beams. The treatment lasted for around 63 minutes, and the total plan monitor units were 27512.38. The dose achieved by organs at risk is tabulated in Table 1.

**Table 1:** OAR doses achieved in the treatment plan. To aid in context, the max point dose is 1370 cGy for optic pathway - chiasm, right optic nerve, and bilateral optic tract, 1910 cGy for brainstem, and 1170 cGy for bilateral cochlea for two-fraction stereotactic radiosurgery, as per Timmerman [6]. OAR: organs at risk.

Organ at risk	D min (cGy)	D mean (cGy)	D max (cGy)
Brainstem	21.06	84.66	305.91
Chiasm	149.84	484.57	D_0.03cc _814.29, Max point dose 1277.05
Cochlea, left	178.45	210.16	223.66
Cochlea, right	36.82	47.61	65.64
Eye, left	44.87	344.99	1719.41
Eye, right	16.24	25.51	68.09
Lens, left	85.41	130.03	186.71
Lens, right	16.97	20.79	47.14
Normal brain	15.42	59.06	2076.15
Optic nerve, right	23.92	58.47	195.56
Optic tract, left	50.92	196.89	305.58
Optic tract, right	80.69	191.85	358.21
Pituitary gland	201.41	435.37	1085.69

**Figure 3:**
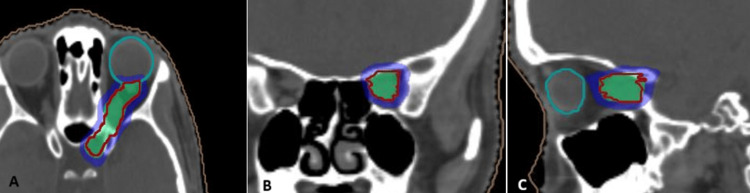
Axial (A), coronal (B), and sagittal (C) sections of the dose distribution showing 15 Gy isodose in green and 7.5 Gy isodose in blue. The target is delineated in red, left eyeball is delineated in cyan.

**Figure 4:**
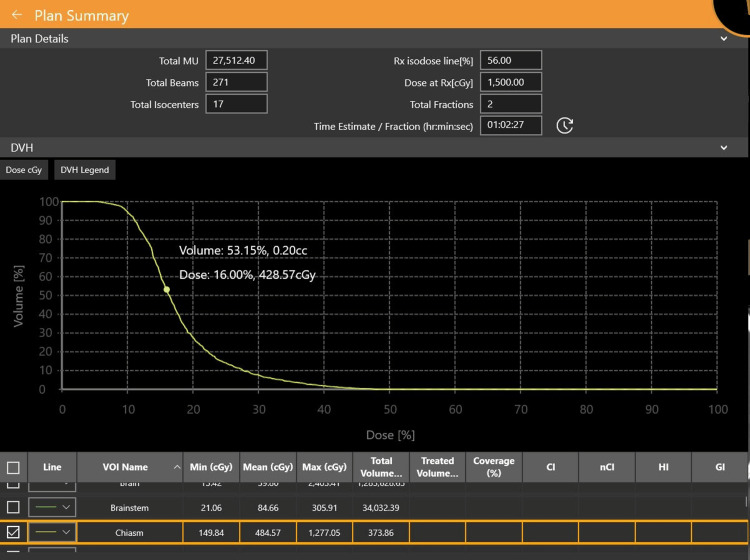
Dose volume histogram showing the dose received by the chiasm.

The isodose distribution of the approved plan is shown in Figure 3, and the dose volume histogram showing the dose received by the chiasm is shown in Figure 4.

The patient did not report any treatment-related side effects seven days after radiosurgery. One month post radiosurgery, clinical review of the patient showed persistent diminished vision in the left eye and intact vision in the right eye (6/6). The patient also gave a history of on-and-off headaches, not requiring any medication. Further follow-up is slated at three, six, 12, and 18 months post treatment, and yearly thereafter with clinical evaluation, ophthalmology evaluation, and MRI studies.

## Discussion

The optic nerve, being an eloquent area, has precluded tissue diagnosis in our case. However, advanced neuroimaging has greatly enabled to make accurate diagnosis. This is backed by the study by Pepin et al., who in 2006 had reviewed anterior OPGs and concluded that advanced neuroimaging has nearly always obviated the need for biopsy [4]. Sawamura et al. proposed that in cases that are clinically or radiologically typical, surgical biopsy is frequently not required, and curative resection is seldom accomplished when the functional outcomes for patients are given serious consideration [8].

OPGs are predominantly a childhood tumor, with the age of presentation of less than six years, whether or not they are associated with NF1. Consequently, most of the available studies have mentioned the use of chemotherapy in children as a cautious approach to delay the side effects of radiotherapy during the developmental age. With the advancement of radiotherapy delivery techniques, especially with radiosurgery, the side effect profile, that too in an adult, needs to be reassessed. The consensus statement from the NF1 Optic Pathway Glioma Task Force in 2004 acknowledged that radiotherapy has the longest experience with the treatment of OPGs and addressed the pros and cons of radiotherapy [9]. They observed that chemotherapy may delay tumor growth, but as the tumor ultimately progresses, the need for a more definitive treatment remains inevitable. Radiotherapy is cited as a treatment modality in disease progression as a means to arrest tumor growth. They concluded that there is less data to support the side effect profile of radiotherapy, and the concerns, though valid, are mostly theoretical.

Extensive research was conducted before arriving at a treatment approach, keeping in mind the patient’s late presentation with a progressing disease. We found ourselves facing a question: Can radiation be preferred as the choice of treatment since the patient is over the critical age and brain cell division is largely complete (20 years at presentation in our case)?

We came across an article published in 2004, wherein late-onset optic pathway tumors in children with NF1 were studied [10]. The article provided insights on eight patients with late-onset OPG (older than six years) across three centers. Three out of eight children were treated for OPG with chemotherapy, aged 10.5, nine, and six years, respectively. It is worth noting that all three patients were diagnosed with NF1 at the ages of three, nine, and three years, respectively. Case 7 of the article matched with our patient profile (F/Caucasian, presented at the age of 22 years with a concurrent diagnosis of NF1 and OPG but with stable vision). This patient was treated with radiotherapy at the age of 26 due to disease progression. Dose and fractionation, however, were not mentioned. The reason for not considering chemotherapy was also not mentioned.

The largest available studies pertaining to SRS for OPG were done using the Gamma Knife (GK) radiosurgical system. A systematic review was published by Rashidi et al. earlier this year, which sheds light upon seven studies using GK as the radiation source for optic/hypothalamic pathway glioma [11]. The study observed that the therapeutic dose administered was in a range of 11 to 15 Gy in one to four fractions. A meta-analysis and systematic review on repeat SRS for vestibular schwannoma after previous SRS observed that a dose of 11 Gy or less is considered subtherapeutic and may lead to recurrence [12]. These findings can be extrapolated in the case of OPG as they share the same radiobiology.

El-Shehaby et al. in 2016 had evaluated the safety and efficacy of single-session SRS for optic pathway/hypothalamic gliomas [13]. The rationale behind this study stemmed from the fact that both the target tissue (low-grade glioma in this case) and the surrounding normal tissues are radiobiologically the same (late responding tissues). Therefore, fractionation in such a scenario is not expected to produce preferential damage to benign lesions than in the surrounding normal tissues [14]. This, however, if applied in our case, would increase the dose received by the chiasm in a single fraction over and above our constraint, and hence we resorted to a two-fraction regime. For late responding tissues, such as optic nerve glioma, as well as our surrounding normal CNS tissues, the α/β ratio is assumed to be 3. For a total dose of 15 Gy and a dose per fraction of 7.5 Gy (two fractions), the biologically effective dose (BED) is 52.50 Gy and equivalent dose in 2 Gy fractions (EQD_2_) is 31.50 Gy, whereas for a single fraction, the BED is 90 Gy and EQD_2_ is 54 Gy for both optic nerve and chiasm. Thus, by employing fractionation, we were able to give a therapeutic dose to the disease while mitigating the dose received by the surrounding normal structures.

A case report on fractionated stereotactic radiotherapy (FSRT) using CyberKnife (CK) as definitive treatment in a sporadic case of optic nerve glioma in an 11-year-old child was published by Uslu et al. in 2013. The treatment was carried out with 21 Gy in five fractions delivered to the 83% isodose line [15]. The BED was 50.40 Gy, and EQD_2_ was 30.24 Gy in this case, for an α/β ratio of 3. Taking the aforementioned studies into consideration, we arrived at the dose of 15 Gy in two fractions (BED is 52.50 Gy and EQD_2_ is 31.50 Gy). Head-to-head comparison of the above-mentioned case report with our study shows a more conformal dose distribution and subsequent decrease in dose received by organs at risk in the Zap-X plan. This may be attributed to the fairly low dose bath with Zap-X GRS, as compared to other radiosurgical systems, such as CK. A literature review regarding the use of CK was published in 2025 by Donati et al., which explored CK’s potential for effective tumor control with favorable toxicity profiles in pediatric tumors of the skull and head and neck region. However, a potential “low dose bath” effect warrants a risk and benefit-weighted approach [16].

Proton beam therapy (PBT) is considered to be the benchmark for precision radiotherapy, especially in pediatric oncology, owing to its unmatched conformity to target volumes and steep dose gradients, thus leading to substantial normal tissue sparing. In a dosimetric comparative study on proton radiation therapy for pediatric OPGs, comparison with 3D planned conventional photons and a standard photon technique substantiates this claim [17]. Indelicato et al., in 2019, published a study in which 174 children with low-grade glioma were prospectively treated with PBT between 2007 and 2017 [18]. Clinical outcomes, toxicity, and patient, tumor, and treatment-related variables were analyzed after a median follow-up of 4.4 years. The study encountered pilocytic astrocytoma (47%) as the most common histology and diencephalon/optic pathway (52%) as the most common tumor subsite. Patients achieved a 91% local control rate and 90% progression-free survival rate, who were treated to a dose of 54 GyRBE. Of the patients, 32% developed pseudoprogression. Post-radiation follow-up revealed that 22% of patients developed new-onset central hormone deficiency (grade 2 toxicity). Of these 39 patients, 37 had supratentorial midline tumors involving the optic pathway, thalamus, and/or tectum. The most common hormone affected was growth hormone, which was deficient in 31 out of 39 patients. One patient diagnosed with OPG experienced significant permanent visual impairment due to retinopathy. Other toxicities include grade 2 acute gastrointestinal toxicity (12.6%), grade 1 asymptomatic vasculopathy (34%), and grade 2 headache (1.1%).

Table 2 provides a summary of relevant studies concerning radiotherapy for OPGs.

**Table 2:** Summary of relevant studies concerning radiotherapy for optic pathway gliomas. NF1: neurofibromatosis type 1; OPG: optic pathway glioma; RT: radiotherapy.

Study	Radiotherapy modality	NF1 status	Patient cohort	Treatment details	Outcomes	Key findings
Uslu et al. (2013) [15]	Fractionated stereotactic radiotherapy (FSRT)	Sporadic	Case report, 11 years old	21 Gy in 5 fractions using CyberKnife	Tumor regression from 20 × 17 mm to 13 × 10 mm; vision preserved without severe toxicity	Demonstrates the feasibility of FSRT in treating optic nerve gliomas
Rashidi et al. (2025) [11]	Gamma Knife radiosurgery	Mixed (NF1 and sporadic)	Systematic review	Various dose regimens across studies	Tumor control rate ~90%; vision stable or improved in most cases	Effective in both NF1 and sporadic cases; high tumor control and vision preservation
El-Shehaby et al. (2016) [13]	Single-session Gamma Knife radiosurgery	Majority sporadic	22 patients	8–14 Gy, single session	Tumor shrinkage in 12, stable vision in 12, and improved vision in 6	Safe and effective; careful dosing needed due to proximity to optic structures
Robert-Boire et al. (2017) [1]	Not specified	42.5% NF1	40 patients	7 patients received radiotherapy, details not specified	NF1 patients presented earlier, better visual prognosis	NF1 cases tend to present earlier but are less aggressive
Awdeh et al. (2012) [19]	Conformal radiotherapy	Included both NF1 and sporadic	20 children	~54 Gy	Long-term visual stability in most cases	Use of chemotherapy prior to radiotherapy decreased visual acuity compared to upfront primary radiation therapy.
Hanania et al. (2021) [20]	Proton beam therapy (PBT)	Sporadic	38 children	50.4 GyRBE median	Vision improved/stable in 90%; mild toxicity	Early RT can be used as initial/first-line salvage therapy for sporadic cases, preserving vision
Listernick et al. (2004) [10]	Not specified	All NF1, late-onset OPGs	8 patients	1 patient received radiotherapy, details not specified	Continued monitoring is recommended to detect late-onset or late-progressive OPGs	Highlights the need for long-term surveillance in older children with NF1

This case report appears to be the first published data regarding the use of GRS in optic nerve glioma, to the best of our knowledge. The lower incidence of optic nerve gliomas, coupled with the novelty of the Zap-X GRS system, can be cited for the lack of published data pertaining to our study. However, a dosimetric comparison of ZAP-X, GK, and CK SRS for single brain metastasis has revealed superior conformal dose distribution and better protection for brain tissue with the higher quality plans generated using the Zap-X treatment planning software [21]. Thirteen patients with solitary brain metastasis treated with CK were retrospectively replanned using the ZAP-X plan system and the GK ICON plan system with the same prescription dose and organs at risk (OARs) constraints. The prescription dose of planning target volume was normalized to 70% for both ZAP-X and CK, while it was 50% for GK. The treatment time was shorter and the total monitor units were less compared to CK plans, while the Paddick conformity index (CI) and gradient score index (GSI) were higher, and Paddick gradient index (GI) was comparable to the GK plans, which is frequently referred to as the “gold standard” in SRS. Additionally, Zap-X enables a bunker-less treatment and non-requirement of constant source replenishing (when compared to GK), which eventually cuts down on the cost without compromising the quality of treatment. This offers an advantage to the pediatric oncology population, who are long-term survivors, to receive high-end treatment at a cost, weighing the risk of late toxicity and secondary malignancy.

## Conclusions

Radiotherapy has come a long way owing to advanced technology in delivering precise radiation that would limit the dose delivered to the surrounding structures. CyberKnife robotic radiosurgery system, PBT, and the Zap-X gyroscopic radiosurgical system are the front runners in delivering such precise radiation. No upfront clinical trial has been conducted to study the efficacy of radiosurgery delivered through such an advanced system compared to chemotherapy. So, the potential of using radiosurgery as a first-line treatment in OPG is yet to be fully understood. If treated early, radiotherapy can be delivered to as minimal a target volume as required, which may result in a lesser occurrence of vision loss, risk of precocious puberty, growth hormone deficiency, cerebrovascular complications, and secondary neoplasms.

The present study has been able to successfully demonstrate favorable use of GRS owing to its dosimetric superiority and early clinical patient experience. To answer the question, "Can radiation be preferred as the choice of treatment in a patient who is over the critical age and brain cell division is largely complete?", our findings suggest it is plausible to hypothesize that "Early intervention with gyroscopic radiosurgery (GRS), CyberKnife, or proton beam therapy in patients with optic pathway gliomas (OPGs) may result in superior vision preservation and reduced endocrinological and vascular complications compared to conventional treatment modalities". However, long-term follow-up is advocated to assess the prolonged effects of treatment on vision and hormonal status, as well as late toxicity, if any. Multi-institutional studies involving a larger cohort of patients are required to comment on the safety and efficacy of SRS in optic nerve glioma.
